# Intensive follo w-up after liver resection for colorectal liver metastases: results of combined serial tumour marker estimations and computed tomography of the chest and abdomen – a prospective study

**DOI:** 10.1038/sj.bjc.6603219

**Published:** 2006-06-27

**Authors:** S Bhattacharjya, R Aggarwal, B R Davidson

**Affiliations:** 1 Department of Surgery, Royal Free and University College School of Medicine, University College London, The Royal Free Hospital, Pond Street, London NW3 2QG, UK

**Keywords:** colorectal liver metastases, follow-up, tumour markers, imaging

## Abstract

The aim of the study was to prospectively evaluate an intensive follow-up programme using serial tumour marker estimations and contrast-enhanced computed tomography (CT) of the chest and abdomen in patients undergoing potentially curative resection of colorectal liver metastases. Seventy-six consecutive patients having undergone potentially curative resections of colorectal liver metastases in a single unit were followed up with a protocol of 3 monthly carcinoembryonic antigen and carbohydrate antigen 19-9 estimations and contrast-enhanced spiral CT of the chest, abdomen and pelvis for the first 2 years following surgery and 6 monthly thereafter. The median period of follow-up was 24 months (range 18–60). Recurrent tumour was classed as early if within 6 months of liver resection. Thirty-seven of the 76 patients (49%) developed recurrence on follow-up. Nineteen recurrences were in the liver alone (51%), 16 liver and extrahepatic (43%) and two extrahepatic alone (6%). Of the 19 patients with isolated liver recurrence, eight developed within 6 months of liver resection none of which were resectable. Of the 11 recurrences after 6 months, five (45%) were resectable. Of the 37 recurrences, CT indicated recurrence despite normal tumour markers in 19 patients. Tumour markers suggested recurrence before imaging in 12 and concurrently with imaging in 6. In the 12 patients who presented with elevated tumour markers before imaging, there was a median lag period of 3 months (range 1–21) in recurrence being detected on further serial imaging. Seventeen patients who developed recurrence had normal tumour markers before initial resection of their liver metastases. Of these 17, 10 (58%) had an elevation of tumour markers associated with recurrence. Over a median follow-up of 2 years following liver resection, the use of CT or tumour markers alone would have failed to demonstrate early recurrence in 12 and 18 patients respectively. A combination of tumour markers and CT detected significantly more (*P*<0.05) recurrence than either modality alone. Tumour markers and CT should be used in combination in the follow-up of patients with resected colorectal liver metatases, including patients whose markers are normal at the time of initial liver resection.

The incidence of colorectal cancer in the Western World is approximately 50 per 100 000 with a cumulative lifetime risk of approximately 5% (Rudy and Zdon, 2000; Malafosse *et al*, 2001; McCormick *et al*, 2002). Liver metastases are one of the principal causes of death in these patients. About 25% of patients have liver metastases at the time of presentation and another 20% subsequently develop liver metastases (Norum *et al*, 1997; Lyass *et al*, 2001). Median survival of untreated colorectal liver metastases is approximately 9 months with few survivors beyond 2 years (Bengtsson *et al*, 1981; Fujimoto *et al*, 1985; Giacchi *et al*, 1988; Daly *et al*, 1989). The reported 5-year survival following surgical resection of liver metastases is between 35 and 40% (Scheele *et al*, 1990; Ballantyne and Quin, 1993; Leow *et al*, 1997; Beard *et al*, 2000; Bolton and Fuhrman, 2000; Ruiz *et al*, 2000; Weber *et al*, 2000; Yamaguchi *et al*, 2000; Ruers and Bleichrodt, 2002).

A variety of imaging strategies have been suggested in the follow-up of patients after resection of colorectal liver metastases (Tartter *et al*, 1981; Fantini and DeCosse, 1990; Baulieu *et al*, 2001; Glover *et al*, 2002; Longo and Johnson, 2002). The presence of recurrent liver metastases was historically considered to have a poor prognosis and routine screening for patients having undergone resection of colorectal liver metastases was felt not to be justified by analysis of cost to benefit ratio (Biggs and Ballantyne, 1994; Beard *et al*, 2000). However, recent studies have shown a 35–40% survival at 5 years following surgery for recurrent liver metastases (Tuttle *et al*, 1997; Yamamoto *et al*, 1999a; Muratore *et al*, 2001; Suzuki *et al*, 2001; Yamada *et al*, 2001a). These results reflect a better outcome with a more aggressive approach towards the treatment of colorectal liver metastases and careful follow-up of patients following resection of the liver metastases is therefore essential.

The main follow-up options for patients with resected colorectal liver metastases combine serial estimations of tumour markers with imaging by ultrasound, computed tomography (CT), magnetic resonance imaging or position emission tomography (PET) scan. Spiral CT has a high accuracy in the detection of colorectal liver metastases (Bhattacharjya *et al*, 2004). This prospective study presents the results of using serial tumour marker estimation in conjunction with contrast-enhanced CT of the chest, abdomen and pelvis in the follow-up of patients following liver resection for colorectal metastases.

## MATERIALS AND METHODS

Prospectively collected data on 76 patients who had undergone potentially curative resection for colorectal liver metastases in a single centre over a 5-year period from January 1996 to December 2001 was reviewed. During this period 120 patients were referred for consideration of liver surgery for metastases following a curative resection of a colorectal cancer. Staging investigations over this time period included contrast CT of the chest, abdomen and pelvis, a dynamic gadolinium enhanced magnetic resonance of the liver and a CT arterial portography. At surgery these patients were further assessed with intra-operative ultrasound and bimanual palpation. The results of this staging algorithm have been previously described (Bhattacharjya *et al*, 2004).

Eighty-nine patients had resection of their liver metastases. Of these 13 were excluded from the current follow-up study owing to noncurative liver resections (margin positive *n*=4) or incomplete follow-up (*n*=9). All 76 patients had tumour marker estimation before liver resection and at 6 weeks following surgery. The patients entered a follow-up protocol of measuring serum carcinoembryonic antigen (CEA) and carbohydrate antigen 19-9 (CA 19-9) plus contrast-enhanced spiral CT (CECT) of the chest and abdomen at 3 monthly intervals for the first 2 years and thereafter at 6 monthly intervals till 5 years. The imaging protocol for the contrast CT of the chest and abdomen during follow-up was the same as used for pre-operative staging and is outlined below. The follow-up imaging was carried out at the Royal Free Hospital. The minimum period of follow-up was 18 months, median 24 months.

### Technique of CECT of the chest

Contrast-enhanced axial images of the chest were obtained on a GE HiSpeed spiral CT with 10 mm collimation at 10–15 m s^−1^ table speed 20 s following injection of 75–100 ml Omnipaque 300 (Nycomed, Amersham Health, Little Chalfont, Bucks, UK) through a peripheral vein. Well-defined nonenhancing lesions of soft tissue density in the lung parenchyma were considered to be suspicious and were followed up by either fluoro-2-deoxy-D-glucose (FDG) PET scan or repeat CECT of the chest at 6 weeks. Lesions that showed uptake of FDG or had increased in size were classified as pulmonary metastases.

### Technique of CECT of the abdomen

Portal phase images were obtained 70 s following injection of 75–100 ml Omnipaque 300 through a peripheral vein infused at a rate of 4 ml/s^−1^ on a GE HiSpeed spiral CT with 7–10 mm collimation at 10–15 mm s^−1^ table speed. The abdominal scans were performed from the level of the domes of the diaphragm to the symphysis pubis. Continguous 7- to-10-mm-thick axial images were reconstructed from the volumetric data. Well-defined nonenhancing water density lesions were considered to be benign. Iso- or hypodense lesions with rim enhancement were considered to represent metastases. Consultant radiologists with an interest in hepatobiliary radiology reviewed the images. An ordinal category system was used to group the patients with liver metastases into solitary metastases H1, 2–5 metastases H2 and >5 metastases H3.

Carcinoembryonic antigen and CA19-9 levels were assessed in the serum by immunoassay techniques performed on the Roche Analyser E170 (Roche Diagnostics, Lewes, East Sussex UK). Serum CEA levels greater than 5 ng ml^−1^ and CA 19-9 levels greater than 37 U ml^−1^ were considered abnormal. Tumour marker levels were correlated with findings on imaging and clinical follow-up of the patients. The effect of the following variables on disease-free survival was assessed: Duke's stage of bowel cancer, tumour marker levels, number of liver metastases and adjuvant chemotherapy for the primary colon cancer.

A cost analysis of the protocol based on costs for private investigations in an NHS hospital was performed. The cost of the follow-up surveillance programme was evaluated based on an estimated cost of a spiral CT at £500.00 and cost per CEA and CA19-9 estimation at £5.00 per test. The cost of clinician follow-up time was not analysed.

Statistical analysis was performed using SPSS version 10. Survival was assessed using Kaplan–Meier curves and compared by the log-rank test. *χ*^2^ and where appropriate Fishers exact test were used. Statistical significance was considered to be present on *P*<0.05.

## RESULTS

Seventy-six consecutive patients who had undergone liver resection for colorectal liver metastases were analysed in this follow-up study. The median age was 60 years (range 29–77) with a male-to-female ratio of 40 : 36. Fifty of the 76 patients presented with synchronous metastases, whereas 26 had metachronous metastases. Forty-two had a node negative and 34 a node-positive primary tumour. All primary resections had clear histological resection margins. Thirty patients had a solitary metastasis, 44 had 2–5 and 2>5 metastases (Table 1). Nineteen patients underwent a right or left hepatectomy, 13 an extended right or left hepatectomy and 38 a segmental (*n*=20) or nonanatomical wedge hepatic resection (*n*=18). Five patients had an extended hepatectomy plus wedge resection (Table 2).

Results of CT scans were available for all 76 patients and there were a total of eight scans per patient during the median 24 month follow-up (range of follow-up 18–60 months). Fifty-four patients were followed up at our centre exclusively and eight results for tumour marker estimations were available for each of these patients during the follow-up period. Twenty-two patients were followed up jointly with their referring hospitals. In these 22 patients, the tumour marker estimation was performed locally and a median of 6 (range 4–8) results was available for this group.

No recurrence was detected in 39 patients over the follow-up period. Thirty-seven patients had a recurrence. Fifteen presented with recurrence within 6 months of the liver resection and 22 more than 6 months following liver resection (Table 3). Nineteen had recurrence in the liver only, 13 liver and lungs, three liver and hilar and celiac lymph nodes and two had extrahepatic recurrence (Table 3). Recurrence following liver resection was not influenced by the Dukes stage of the primary bowel cancer, 20 of the 41 patients with a Dukes B cancer developed recurrence (49%) and 17 of the 34 with a Dukes C stage cancer (50%). Of the 20 recurrences who had Dukes B cancers, six recurred within and 14 after 6 months of liver resection. Of the 17 Dukes C recurrences nine recurred within 6 months and eight after 6 months. Delayed recurrence was more common in the Dukes' B group (14 out of 20, 70%) than the Dukes' C group (eight out of 17, 47%), although this was not statistically significant.

Of the patients with recurrent metastases, 15 underwent initial resection for solitary liver metastases (H1), 21 2–5 metastases (H2) and 1>5 metastases (H3). Nine patients had a right or left hepatectomy, seven an extended hepatectomy, 18 had a wedge resection and three an extended hepatectomy plus wedge resection for their liver metastases at initial presentation (Table 4). No association was observed in the type of resection performed or number of tumours (H stage) and the timing of recurrence being detected.

Of the 76 patients included in this study, 65 had raised (secreting tumours) and 17 had normal (nonsecreting tumours) tumour markers before the first liver resection. In the 37 patients with recurrences, 12 (group 1) had raised tumour markers before CT evidence; six (group 2) had tumour markers correlating with CT evidence and 19 patients (group 3) had CT evidence of recurrence before elevation of tumour markers. In group 1, five patients had elevated tumour markers at the time of initial liver resection and had therefore, secreting tumours. In groups 2 and 3, three and 12 patients had secreting tumours respectively (Table 5). In total, 20 of the 37 patients with recurrences had secreting tumours and in them elevated tumour markers were predictive of recurrence in eight (40%). Seventeen patients did not have a secreting tumour and in 10 (59%) elevated tumour markers on follow-up were predictive of recurrence. Raised tumour markers were the first indication of recurrent disease in 18 of 76 patients (24%). Normal tumour marker levels with CT evidence of recurrence were noted in 19 of 76 patients (25%). A protocol of using both tumour markers and CT in the postoperative period significantly improved the diagnostic accuracy and detected recurrent disease in 37 of 76 patients (49%) (*P*<0.05).

Of the 12 patients with rise in tumour markers and normal CTs, CEA was elevated in eight and CA 19-9 in nine (both in 5). Of the six where tumour markers and imaging both suggested a recurrence five patients had an elevated CEA and five elevated CA 19-9. In the 12 patients with elevated tumour markers with normal CT, there was a median lag period of 3 months (range 1–21 months) before recurrence was detected on further imaging.

Nineteen patients had recurrent metastases in the liver alone. Eight (42%) presented within 6 months of surgery. None of these had resectable disease on repeat staging. Eleven (58%) presented after 6 months, of whom five (45%) were resectable. Four of these patients underwent a further liver resection. One patient refused further surgery. One of the patients undergoing a repeat hepatic resection developed a further recurrence at 18 months. The other three patients have remained well and disease-free (median follow-up of 30 months (range 12–54 months)). Of the 53 patients who had received chemotherapy before the initial liver resection, 26 (49%) developed recurrent disease (18 Dukes' B and 8 Dukes' C). Of the 23 patients who did not receive initial chemotherapy, 11 (49%) developed recurrence.

The cost of the current protocol that identified five out of 76 patients who could have been potentially cured by repeat liver resection was £310 080.00 over a 2-year period (cost per CT scan=£500.00; cost per CEA and CA19-9 estimation at £5.00 per test; total of (76 × 8) 608 scans and blood tests). Therefore, the cost per patient potentially cured per year was £31 000. The cost of following up an individual patient by this protocol was £2040.00 per year.

## DISCUSSION

Intensive follow-up programmes to screen for recurrent cancer should be designed to detect disease in a selected population where a therapeutic intervention altering ultimate prognosis and survival can be offered. Where this is not possible, these tests unnecessarily enhance patient anxiety and may reduce their overall quality of life. A selective approach is often required to justify the costs of screening, which has an unacceptable cost–benefit ratio when applied universally (Beard *et al*, 2000; Gazelle *et al*, 2003).

The surveillance programme used in this study of patients who had undergone resection of colorectal liver metastases detected recurrent disease in 49% of patients. Of these recurrences, 51% were in the liver alone, 43% liver and extrahepatic and 6% extrahepatic alone. This incidence and pattern of recurrence is similar to data previously published (Sugihara *et al*, 1993) and confirms that the majority of patients developing recurrence develop liver- alone disease, which could potentially be amenable to further liver surgery (Topal *et al*, 2003). Where surgery is not possible, alternate therapies like radiofrequency ablation may provide local tumour control (Gillams and Lees, 2005).

None of the patients with recurrent liver metastases detected within 6 months of their liver resection had resectable disease. These early recurrences may represent microscopic metastatic disease or occult metastases present at the time of liver resection that only becomes apparent under the trophic effects of liver regeneration. Local recurrence owing to incomplete resection of the liver metastases is unlikely as patients with histologically involved liver resection margins were not included in this follow-up study. The ability to detect occult disease is limited by the technical resolution of currently available imaging equipment. Intra-operative ultrasound is the most sensitive technique and all these patients had had an intra-operative ultrasound at the time of first liver resection (Machi *et al*, 1987; Agrawal *et al*, 2006). As none of these patients proved to be amenable to further liver resection they will represent a poor prognosis group in whom surveillance is less likely to be beneficial.

Of the patients who developed liver metastases after 6 months, a further curative resection was possible in 45%. The reported 5-year survival in patients undergoing repeat resections of their liver metastases is in the region of 35–40% (Yamamoto *et al*, 1999a; Beard *et al*, 2000; Muratore *et al*, 2001; Suzuki *et al*, 2001; Yamada *et al*, 2001a; Gazelle *et al*, 2003) and therefore a follow-up aimed at detecting these resectable recurrences is useful and should be instigated at 6 months.

On follow-up the incidence of recurrent liver metastases following liver resection in patients whose colorectal cancer was Dukes' B and C was similar (49 *vs* 50%). Recurrence after 6 months was higher in patients with Dukes' B cancer, although the difference was not statistically significant. A predominance of late recurrences in the less-advanced Dukes stage may have been anticipated and the lack of significance may be owing to the small number of patients in the study and requires to be validated in a larger series. The Dukes stage of the colon cancer is a factor influencing outcome in multivariate analysis of first liver resection (Fong *et al*, 1999). In this study, the Dukes stage did not influence the recurrence rate following liver resection. No association was observed between the number of tumour nodules and frequency of recurrence. Although the presence of more than a single metastasis has been reported to be associated with a poorer overall outcome with liver resection (Fong *et al*, 1999; Muratore *et al*, 2001; Yamada *et al*, 2001b), whether it is the absolute number of metastases or the total volume of metastatic disease that affects outcome is not known (Muratore *et al*, 2001). In the current study, the outcome of solitary and multiple liver metastases in terms of disease recurrence was similar, supporting the role of liver resection in patients with multiple liver metastases. Similar results have been reported in the literature (Ambiru *et al*, 1999; Fong *et al*, 1999; Yamamoto *et al*, 1999b).

The incidence of nonsecretory colorectal cancers is in the order of 20% (Duffy, 2001; Carpelan-Holmstrom *et al*, 2002). In this study, 17 of 76 (22%) patients with colorectal liver metastases had normal tumour markers. The mechanism underlying expression of CEA in patients with liver metastases whose primary cancers did not express CEA are unclear. Possible explanations include the degree of differentiation of the primary tumour, effects of transforming growth factor*α*, tumour/host lymphocyte interactions causing hepatocyte apoptosis or a loss of hepatic first-pass effect (Duffy, 2001). An important finding in this study is that 60% of the patients who had normal tumour markers before resection of their liver metastases had elevated tumour markers with recurrence. This would suggest that serial tumour marker assessments should be performed in all patients during follow-up irrespective of the levels before resection of the metastases. While the role of CEA in screening following resection of colorectal liver metastases is well-documented (Hohenberger *et al*, 1994; McCall *et al*, 1994; Paganuzzi *et al*, 1994; Lucha *et al*, 1997; Novell *et al*, 1997; Hocking and Morris, 1998; Wichmann *et al*, 2000; Ishizuka *et al*, 2001), there is less published evidence on the use of CA 19-9 (Ishizuka *et al*, 2001; Carpelan-Holmstrom *et al*, 2002). In the present study, CEA and CA19-9 were found to be of similar value and complementary in the detection of recurrence following liver resection.

In this study, a protocol of using both tumour markers and CT in the postoperative period improved the detection of recurrent disease. The likelihood of repeat hepatic resection for patients with isolated liver recurrences is less with early recurrences (in this study none) as compared to delayed isolated liver recurrence. This finding needs to be validated in larger studies. Even if early detection does not allow potentially curative resection of recurrent liver metastases, there may be significant clinical benefit to the earlier commencement of systemic chemotherapy or the use of radiofrequency ablation (Elias *et al*, 2004; Gillams and Lees, 2005). The timing of recurrence and its implications for therapy and patient outcome is vital to the design of follow-up protocols.

Cost–benefit analysis must be considered carefully when designing a follow-up screening protocol. This study would suggest that a similar detection of resectable disease could be achieved at lower cost by a protocol of serial tumour marker estimations and CT scans starting at 6 months after initial surgery. This approach, however, assumes that early recurrences (within 6 months) are never resectable and also disregards the possible benefit from the early commencement of chemotherapy or radiofrequency ablation in patients with unresectable disease. The issue of cost–benefit analysis in this context requires further analysis. However, this altered protocol would significantly decrease the costs from £31 008 to £22 542 per patient potentially cured per year (*P*<0.001). Similar costs have been reported with an aggressive approach to colorectal liver metastases that have resulted in an increase in quality of life adjusted years (Gazelle *et al*, 2003).

The design of a follow-up protocol depends on the pattern of recurrent disease. In the present study, recurrence was found in 47% of patients within 2 years of liver resection for colorectal metastases. The initial 2-year period is recognised as being the main-risk period for recurrence (Scheele *et al*, 1995). Disease recurrence occurs in the majority of patients in the liver alone and in a proportion of these a further curative resection may be possible. Our data would suggest that tumours recurring early following liver resection are less likely to be amenable to re-resection. However, this observation was based on a small number of patients and needs to be validated by data from other centres. The pattern of recurrence would support a surveillance programme being more intense in the first 2 years, but the frequency of imaging within this time frame requires further investigation.

In conclusion, this study has demonstrated that both CEA and CA19-9 are useful in the detection of recurrent disease following potentially curative resection of liver metastases and these are complementary to spiral CT. Early recurrences were not amenable to further surgery, but those detected after 6 months were often resectable. The tumour marker/CT follow-up protocol costs were £31K per patient per year.

## Figures and Tables

**Table 1:** Profile of patients undergoing liver resection for colorectal metastases

**Dukes' stage of primary**	**Number of patients**	**Sex M : F**	**Syn.**	**Met.**	**Mean CEA (ng ml^−1^)**	**Mean Ca 19-9 (U ml^−1^)**	**H1 : H2 : H3**
A	1	1 : 0	0	1	5	13	0 : 1 : 0
B	41	23 : 18	22	19	254	666	19 : 21 : 1
C	34	16 : 18	28	6	176	158	11 : 22 : 1

Syn., synchronous colorectal liver metastases; Met., Metachronous colorectal liver metastases; mean CEA, at time of initial liver resection (normal levels 1–5 ng ml^−1^); mean CA 19-9, At the time of initial liver resection (normal levels 17–37 U ml^−1^); H1, solitary metastasis; H2, 2–5 liver metastases; H3, >5 liver metastases.

**Table 2:** Initial liver resections performed

**Dukes' stage**	**R Hemihepatectomy**	**Extended R Hemihepatectomy**	**L Hemihepatectomy**	**Extended L Hemihepatectomy**	**Segment/wedge resection**	**Ext. hemihepatectomy+ wedge**
A					1	
B	10	8	1		19	3
C	5	4	3	2	18	2
						
Total	15	12	4	2	38	5

L, left, R, right.

**Table 3:** Sites of recurrence during follow-up of 76 patients with resected colorectal liver metastases

**Recurrence sites**	**Total no.**	**Recurrence <6/12**	**Recurrence >6/12**
Liver only	19	8	11
Liver+lungs	13	5	8
Liver+lymph nodes	3	1	2
Other	2	1	1

Recurrence <6/12, recurrence diagnosed within 6 months of liver resection; recurrence >6/12, recurrence diagnosed beyond 6 months of liver resection.

**Table 4:** Patterns of recurrence

**Dukes' stage of primary**	**Number of patients**	**No recurrence**	**Recurrence <6/12**	**Recurrence >6/12**
A	1	1	0	0
B	41	21	6	14
C	34	17	9	8

**Number of metastases**	**No. of patients**	**No recurrence**	**Recurrence <6/12**	**Recurrence >6/12**
H1 (<2)	30	15	6	9
H2 (2–5)	44	23	9	12
H3 (>5)	2	1	0	1

**Liver resection**	**No. of patients**	**No recurrence**	**Recurrence <6/12**	**Recurrence >6/12**
R hemihepatectomy	15	8	4	3
Ext. R hemihepatectomy	12	6	4	2
L hemihepatectomy	4	2	0	2
Ext. L hemihepatectomy	2	1	0	1
Segmental or wedge resection	38	20	7	11
Ext. hemihepatectomy+wedge	5	2	0	3

Recurrence <6/12, recurrence diagnosed within 6 months of liver resection; recurrence >6/12, recurrence diagnosed beyond 6 months of liver resection; H1, solitary liver metastasis; H2, 2–5 liver metastases; H3, >5 liver metastases.

**Table 5:** Results of surveillance

**Surveillance result**	**Recurrence <6/12**	**Recurrence >6/12**	**CEA/CA 19-9 elevated before liver resection**
CEA/CA 19-9 raised before CT evidence	5	7	5 (2,3)
CT evidence before raised CEA/CA 19-9	8	11	12 (6,6)
CT and CEA/CA 19-9 correlate evidence	2	4	3 (2,1)

CEA, carcinoembryonic antigen >5 ng ml^−1^; CA 19-9, carbohydrate antigen 19-9 >37 U ml^−1^; CT, computed tomography; recurrence <6/12, recurrence diagnosed within 6 months of liver resection; recurrence >6/12, recurrence diagnosed beyond 6 months of liver resection.
